# Abiotic factors impact on oak forest decline in Lorestan Province, Western Iran

**DOI:** 10.1038/s41598-024-54551-6

**Published:** 2024-02-17

**Authors:** Saeed Mehri, Ali Asghar Alesheikh, Aynaz Lotfata

**Affiliations:** 1 Department of Geospatial Information Systems, Faculty of Geodesy and Geomatics Engineering, K. N. Toosi University of Technology, Tehran, Iran; 2 Department of Pathology, Microbiology, and Immunology, School of Veterinary Medicine, University of California, Davis, USA

**Keywords:** Abiotic stressors, Meteorological factors, Ecosystem sustainability, Drought, Time series, Climate sciences, Ecology, Environmental sciences

## Abstract

The Zagros oak forests in Iran are facing a concerning decline due to prolonged and severe drought conditions over several decades, compounded by the simultaneous impact of temperature on oak populations. This study in oak woodlands of central Zagros forests in Lorestan province analyzed abiotic factors such as climate properties, topographic features, land use, and soil properties from 1958 to 2022. We found that higher elevation areas with steeper slopes and diverse topography show significant potential for enhancing oak tree resilience in the face of climate change. Additionally, traditional land use practices like livestock keeping and dryland farming contribute to a widespread decline in oak populations. Preserving forest biodiversity and ensuring ecological sustainability requires immediate attention. Implementing effective land-use management strategies, such as protecting and regulating human-forest interaction, and considering meteorological factors to address this issue is crucial. Collaborative efforts from stakeholders, policymakers, and local communities are essential to oppose destructive suburban sprawl and other developments. Sustainable forestry practices should be implemented to improve the living standards of local communities that rely on forests and traditional livestock keeping, offer forestry-related jobs, and ensure social security. Such efforts are necessary to promote conservation awareness and sustainable practices, safeguarding this unique and vital ecosystem for future generations.

## Introduction

Forests are the most widely distributed terrestrial vegetation type, and as such, they play a significant role in providing the environmental context for regional and global ecosystems^1,2^. Tree mortality and forest degradation have increased globally during the last few decades. Forest trees are routinely exposed to a wide range of biotic, e.g., fungal infections^3,4^, borer beetles^5^, and green oak tortrix^6^ and abiotic, e.g., extreme climate conditions^7,8^, soil degradation^9^, and topography^10^. These stressors vary in intensity and duration and often occur concurrently or rapidly^11,12^, subjecting forest trees to a constant and diverse range of challenges. Abiotic factors have the potential to amplify the impact of concurrent biotic stress on forest trees, either through direct physiological interactions or by triggering disease outbreaks, ultimately leading to tree decline^13^. With rising temperatures and shifting weather patterns, forests face numerous challenges like deforestation, degradation, and stunted growth^14–17^. These factors, influenced by climate change, undermine the forests’ capacity to serve as crucial carbon sinks^18^.

Droughts profoundly impact trees, leading to considerable stress and an increased risk of tree mortality^17^. During severe drought conditions, the normal functioning of plant cells may be compromised, disrupting the dynamics of both primary and secondary metabolism. Consequently, the trees may experience a reduction in their defense capacity, making them more susceptible to pests and pathogens. The impairment of plant cell functioning under severe drought can weaken the trees’ ability to effectively ward off diseases, further exacerbating their vulnerability to biotic stressors^19–21^. Oak decline is a multifactorial event influenced by several biotic and abiotic (e.g., climate changes and drought) acting in time and space^22–25^.

López-Sánchez et al.^14^, Brown et al.^26^, Hernández-Lambraño et al.^15^, and Macháčová et al.^27^ indicated that climate extremes, e.g., summer drought, precipitation deficit, and high temperatures are the primary causes of oak mortality. Also, there is a complex interplay between climatic parameters and various biotic stressors, highlighting the multifaceted nature of oak decline and the need for targeted conservation efforts to mitigate its adverse effects^26^. Furthermore, Hernández-Lambraño et al.^15^ and Crocker et al.^28^ indicated the risk of oak decline is exceptionally high in mid and upper slopes, south-facing aspects with high solar radiation, and steep convex slope segments with a dry soil moisture regime.

Soil is considered the most important component of forests as it provides minerals and nutrients for plants, and its physical and chemical properties play a crucial role in oak growth^29–32^. Soil sand, silt, and density are important physical properties that affect soil fertility and productivity^33^. Soil physical properties determine the ease of root penetration, water availability and the ease of water absorption by plants, the amount of oxygen and other gases in the soil, and the degree to which water moves both laterally and vertically through the soil. Soil physical properties also influence the natural distribution of forest tree species, growth, and forest biomass production^34^.

**Figure 1:**
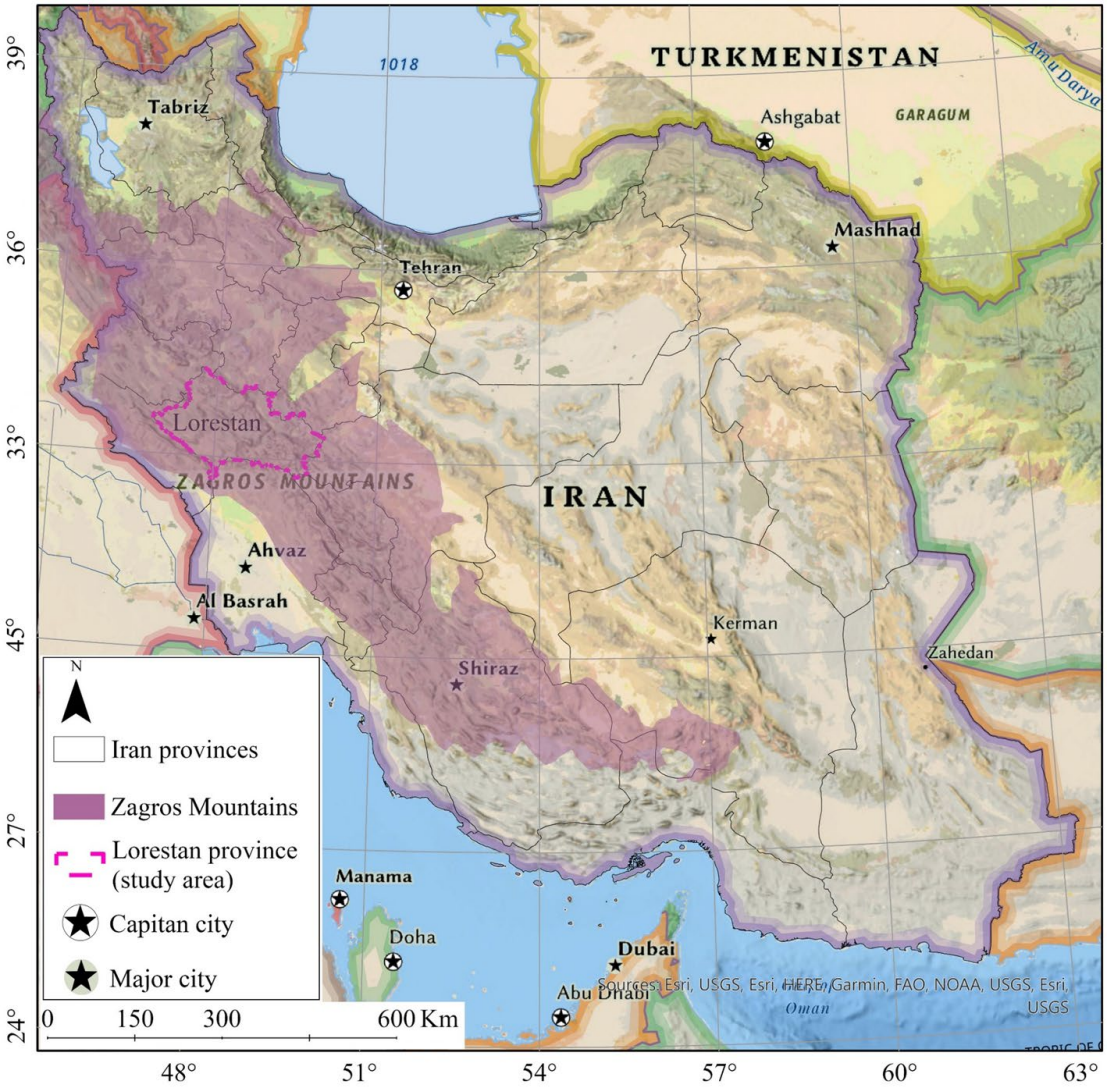
Location of oak woodlands of Zagros forests and study area (Lorestan province), overlaying on National Geographic Style Map in Esri ArcGIS (version 10.8)^45^.

The chemical properties of forest soils are also important for oak forest growth^35^. Important indices of the chemical behavior of all soils are pH, cation-exchange capacity (CEC), Nitrogen, Calcium, Magnesium, Sodium, Soil Organic Carbon (SOC), anion-exchange capacity (AEC), base saturation (BS) percentage, exchangeable sodium percentage (ESP), electrical conductivity, and redox potential^33,35^. Like other oak-dominated woodlands worldwide^10,23–25,36,37^, the Zagros oak forests of Iran have one of the most alarming decline trends^4,37,38^. The Zagros forests cover a large area of the Zagros Mountains ranges, stretching from the northwest to the south of Iran (Fig. 1). Oak, with a diverse range of species, is the dominant genus in this region^38–41^. With an average length and width of 1300 and 200 km, respectively^42^. The Zagros forests cover approximately six million hectares, representing almost 44% of the Iranian forest cover^43^. This is the most significant forest habitat of Iran, distributed across ten provinces of the country, including West Azerbaijan, Kurdistan, Kermanshah, Lorestan, Chaharmahal and Bakhtyari, Kohgiluyeh, and Boyerahmad, Ilam, Khuzestan, Esfahan, and Fars^38,44^. These forests have a Mediterranean climate characterized by warm to hot, dry summers and cold or mild moist winters^16^.

The Zagros forests are dominated by various oak species, creating a diverse ecosystem across the region^39,46^. These oaks are typically classified according to the morphology of their leaves and acorns^38^. According to the classification system of Panahi^47^ and Panahi et al.^48–51^, oaks of the Zagros forests are classified as *Quercus* but in two sections, *Quercus* and *Cerris*, in two groups of lobed-leaved oaks and dentate-leaved oaks. Although most of Lorestan’s oak forests are covered with Persian Oak (*Quercus brantii*)^52–54^, other oak species and their distribution in Loerstan are^38^:

Lobed-leaved oaks (subgenus *Quercus*, section *Quercus*):

- Aleppo oak (*Q. infectoria* subsp. *boissieri* var. *boissieri*)
- Aleppo oak (*Q. infectoria* subsp. *boissieri* var. *pfaeffingeri*)

Dentate-leaved oaks (subgenus *Quercus*, section *Cerris*)

- Brant’s oak (*Q. brantii* var. *brantii*)
- Brant’s oak (*Q. brantii* var. *belangeri*)
- Persian oak (*Quercus brantii* Lindl. subsp. *persica*)

Persian Oak (*Quercus brantii*) is the dominant species in the region as it has more acorn production compared to other species like Alepo oak (*Quercus infectoria*)^55–57^. Furthermore, it is more resilient and tolerant than these other oak species, and its geographic distribution is not limited by elevation or aspect; it is found at various aspects and elevations^58,59^. In addition, it is a low-demanding tree and can grow in different soils. The texture of the soils in Persian Oak habitats varies from clay to clay-loam, and the soil pH ranges between 7.5 and 8.1^60^. The Persian oak is commonly found at lower and mid-elevations, typically between 1000 and 2000 m^61^. Still, the optimal site conditions in the central Zagros are on south-western slopes between 1800 and 2000 m^42,60^.

Moreover, Zagros oak forests are most important in terms of water supply, soil conservation, climate change, and the socioeconomic balance of the entire country^9,62^. Seven first-grade rivers of Iran, carrying about 34.5 billion cubic meters of water, accounting for 45% of the country's groundwater, rise from this region and flow into the fertile plains^38,63^. Therefore, the existence of these water resources is directly dependent upon these oak forests. High ecological potential, especially the possession of rich water supplies, has resulted in a high population density in the region.

In the context of Zagros oak forests in Lorestan, Taghimollaei^64^ reported that soil erosion by wind and water continues to be a fundamental problem in the region, often due to inappropriate cultivation methods and heavy grazing pressure in specific areas. Climatic changes will likely result in greater extremes of drought conditions, which may affect the low-rainfall regions more severely than those with moderate rainfall. Using satellite imagery, Alirezaee et al.^40^ showed that drought stress is the leading cause of oak mortality in Lorestan province. Pilehvar et al.^65^ reported that in the oak woodlands of Lorestan province, oak growth is correlated with altitude, slope, geographical aspects, and the amount of soil organic matter. Badehian et al.^52^ reported oak decline in Lorestan province changed the number of secondary compounds, e.g., total tannin and insoluble sugar in leaves of Presian oak (*Quercus brantii*). Shiravand et al.^66^ showed that from 2000 to 2017, more than 1.7% (42,802 hectares) of oak forests of Lorestan province completely declined.

After 10 years of investigation, Akhtari et al.^67^ reported that the spatial patterns of Persian oak (*Quercus brantii*) in the declined areas of Lorestan’s forests have a clustered distribution. Also, the mean density decreased in declined regions and increased in the non-declined area during 10 years. In addition, there was a significant difference between the tree density and canopy percentage in the regions with and without decline. Shiravand et al.^68^ reported that decline indices had an increasing trend in the oak forests of Lorestan province. Also, there is a growing trend in most decline indices with short-term cycles of 2–4 years of fluctuations on drying indices. Similarly, Shiranvand and Hosseini^44^, using satellite imagery and Geographic Information System (GIS) capabilities, analyzed the oak forests in Lorestan province and showed that land use type and climate factors significantly affect the oak decline.

Early symptoms of oak decline in Zagros oak forests were reported by local experts in 2008^4^. *Charcoal canker* and *Obolarina persica*, fungal diseases, and various pests, such as borer beetles and the green oak tortrix, are the primary causal agents responsible for oak decline^4,5,69^. The common charcoal canker agents are Biscogniauxia species*,* such as *Biscogniauxia mediterranea*^3,4,70^ and *Biscogniauxia rosacearum*^37,71^. Pathogens of this type have always been regarded as secondary fungal invaders that attack only stressed or old trees^23,71^. *Endophytes* live in all of the aerial organs of oak trees (rarely in the leaves), causing no symptoms during its latent phase^72^. This pathogen, however, acts as an opportunistic in conditions of weakened hosts due to abiotic or biotic factors such as prolonged droughts^72,73^. However, there is no consensus regarding how pathogenicity and climate stress interact. Despite some studies suggesting that stress caused by abiotic factors, e.g., drought stress, can increase pathogenicity^18,23,74,75^, others have indicated that these factors reduce the risk of pathogen damage^76^. Consequently, the comprehensive evaluation of abiotic factors in oak decline is still in its early stages of development.

Despite numerous studies on oak decline in the Zagros oak forests^4,23,37,64,65,77–81^, a significant knowledge gap persists concerning the comprehensive evaluation of abiotic factors. Specifically, there is a pressing need for studies primarily focusing on the decline in areas with varying topographic characteristics. Such research would substantially contribute to the existing body of knowledge and offer valuable insights for effective conservation and management strategies in these forest ecosystems.

This study aims to deepen our understanding of oak decline in central Zagros forests in Lorestan province and its relationship with abiotic factors such as climate properties, soil properties, topographic features, and land use. By focusing on these factors, the research seeks to elucidate how they influence the response of forests to climate variability. Furthermore, identifying the temporal behavior of abiotic stress, especially in near real-time, is vital in providing a foundation for effective management and conservation strategies in changing environmental conditions.

## Material and methods

### Study area

Lorestan’s oak forests cover approximately 1.23 million hectares (Fig. 1). Oak woodlands in Lorestan province cover a latitude range of 32°42.96′N to 34°10.79′N and a longitude range of 46°50.36′E to 50°1.19′E. The primary economic activities in this region revolve around livestock and dryland farming^82^. According to official statistics, the Lorestan province has experienced a high increase in livestock keeping. The number of livestock (sum of both small and large livestock) increased from 23,427 in 1971^83^ to 2,191,586 in 2017^84^. In addition, the rainfed farming area in 1979 was 2382 hectares^83^, which increased to 598,078 hectares in 2017^84^.

In Lorestan province, the Zagros oak forests stretch between 341 and 3450 m above sea level. The region receives rain and snowfall primarily due to fronts originating from the Atlantic Ocean, the Mediterranean Sea, and northern Europe, occasionally impacting this area. Precipitation generally decreases from north to south and from west to east. Most of the rainfall occurs during the winter season, with an average ranging between 400 and 800 mm. According to^85^, approximately 70% of the annual rainfall occurs during the latter half of the year. Moreover, the annual mean temperature in this area varies between 9 and 25 °C, depending on the latitude and altitude.

### Data

**Figure 2:**
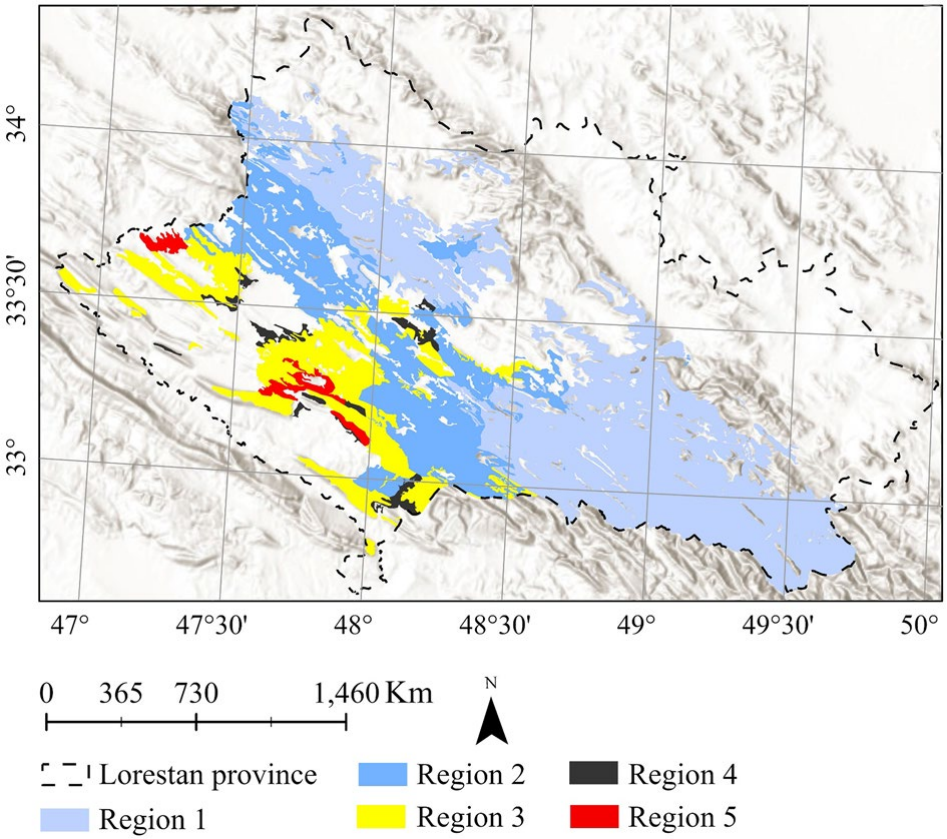
Classification of the study area based on the percentage of declined trees, overlaying on the World Hillshade base map in Esri ArcGIS (version 10.8)^45^.

Eight datasets are used in this paper. First, *the oak decline dataset* was provided by Iran's Forests, Range, and Watershed Management Organization in 2015^9^. In this dataset, oak forests are classified into five regions based on the number of declined trees (Table 1 and Fig. 2).

**Table 1:** Percentage of declined trees in the study area.

Class	% of decline	% of the total area
Region 1	< 1	53.84
Region 2	1 to 25	26
Region 3	25 to 50	16.07
Region 4	50 to 75	2.28
Region 5	75 to 100	1.81

Second*, the elevation dataset* is derived from the SRTM 90 m Digital Elevation Model (DEM), available worldwide with a spatial resolution of 90 m^86,87^. To focus on the specific oak decline regions described earlier, the global dataset was clipped accordingly using ArcGIS’s Extract by Mask toolbox^88^. Subsequently, slope datasets were generated for each decline region at a spatial resolution of 90 m by applying the slope function of ArcGIS software to the elevation dataset^89^.

Third, a *topographic diversity dataset* provides insight into the extent of ecological niches and microhabitats accessible to various species^90^. A description of the topographic diversity index is provided in Appendix A. The data was collected by Conservation Science Partners and is available for free through the Google Earth Engine (GEE) platform^91^. The dataset provides global coverage at a spatial resolution of 270 m.

Fourth, *the maximum and minimum temperatures* used in this study were derived from TerraClimate, a dataset provided by the University of California Merced^92,93^. This dataset employs climatologically assisted interpolation and combines high-resolution climatological normals from various sources, including the WorldClim dataset. The TerraClim dataset, available through GEE (Google Earth Engine), provides a global dataset with 768 monthly observations at a spatial resolution of 4638.3 m. We computed the monthly average maximum and minimum temperatures to establish a time series for each oak decline region. As a result, a comprehensive time series was generated for each region, presenting the average monthly values of maximum and minimum temperatures spanning from 1958 to 2022.

Fifth, *the wind speed* dataset from the University of California Merced covers 1958 to 2022 with a monthly temporal resolution. The dataset has a spatial resolution of 4638.3 m^92,93^. To analyze the wind speed in each oak decline region, we computed the average monthly wind speed individually, resulting in distinct wind speed time series for each region. These time series span from 1958 to 2022, comprising 768 monthly observations for each region’s wind speed.

Six monthly datasets of *evapotranspiration and precipitation* were provided by the University of California Merced from 1958 to 2022 at a spatial resolution of 4638.3 m^92,93^. These datasets were processed to align with the boundary of each oak decline region, and subsequent calculations were performed to generate average monthly values. As a result, a time series comprising 768 values was obtained for each region, capturing the temporal variations of evapotranspiration and precipitation over the specified period.

Seven, *the Palmer Drought Severity Index* (*PDSI*) *dataset*, which also comes from the University of California Merced with monthly temporal resolution^92,93^. The PDSI is one of the most widely used drought indicators addressing two of the most challenging aspects of drought: the intensity of the drought and its beginning and end times^94^. The PDSI index is described in Appendix C. With a spatial resolution of 4638.3 m, the dataset provides global coverage of monthly values from 1958 to 2022. For each region, average monthly values of PDSI are calculated. Consequently, a time series of 768 monthly PDSsI values is created for each region.

**Figure 3:**
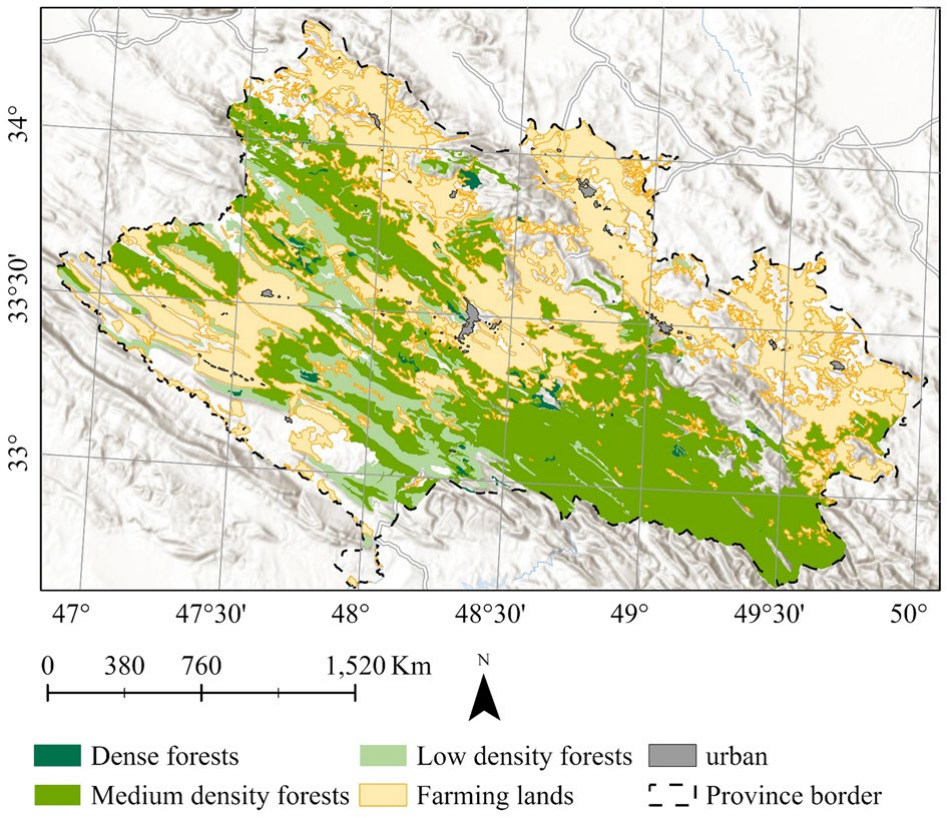
Primary land use classes in the study area, overlaying on the World Hillshade base map in Esri ArcGIS (version 10.8)^45^.

Eight, the *land use dataset* of 2010, which is provided by online repositories^95^. The dataset has a 1:25,000 scale. The primary land use classes include forests, dry farming lands, and urban areas (Fig. 3). Moreover, the dataset classifies forests according to the density of trees into dense, medium, and low forests.

**Figure 4:**
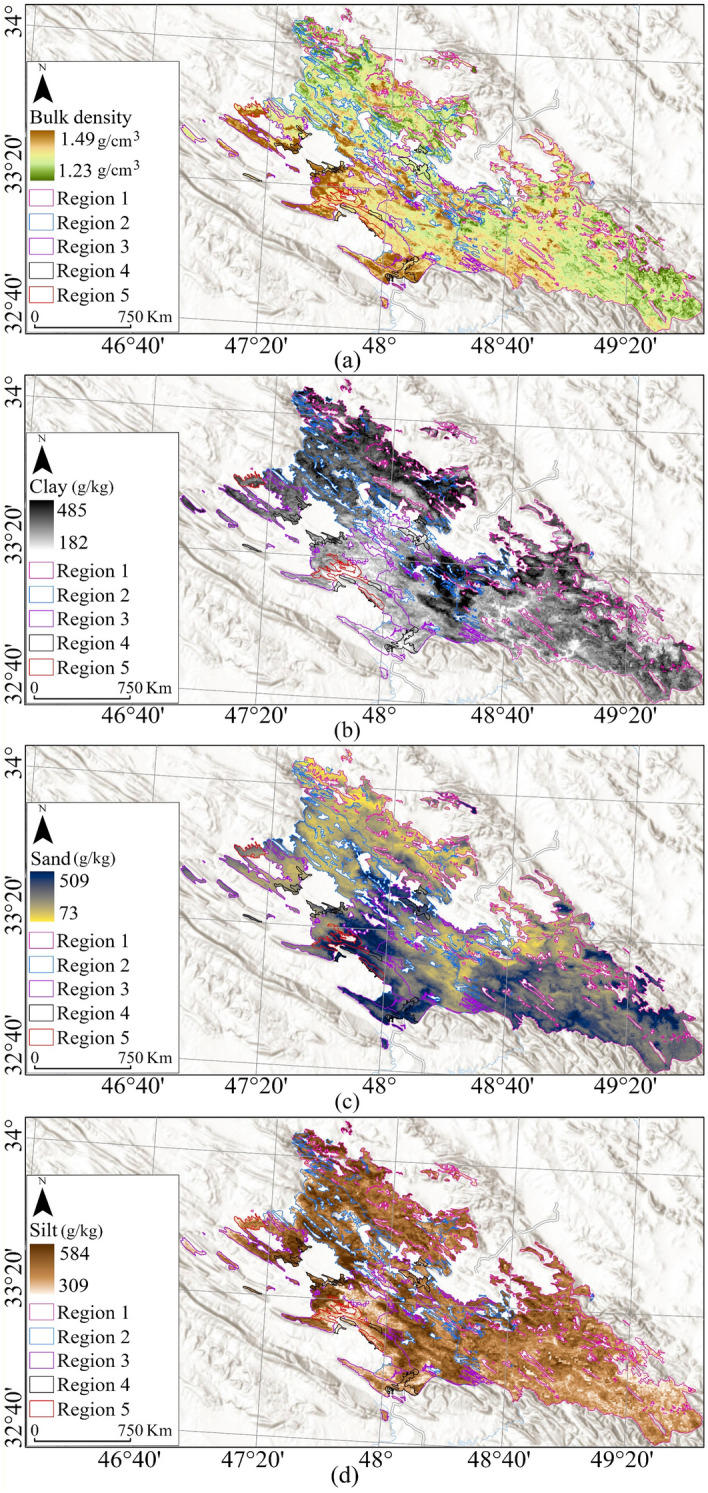
Soil physical properties, Bulk density (**a**), Clay (**b**), Sand (**c**), and Silt (**d**), overlaying on National Geographic Style Map in Esri ArcGIS (version 10.8)^45^.

**Figure 5:**
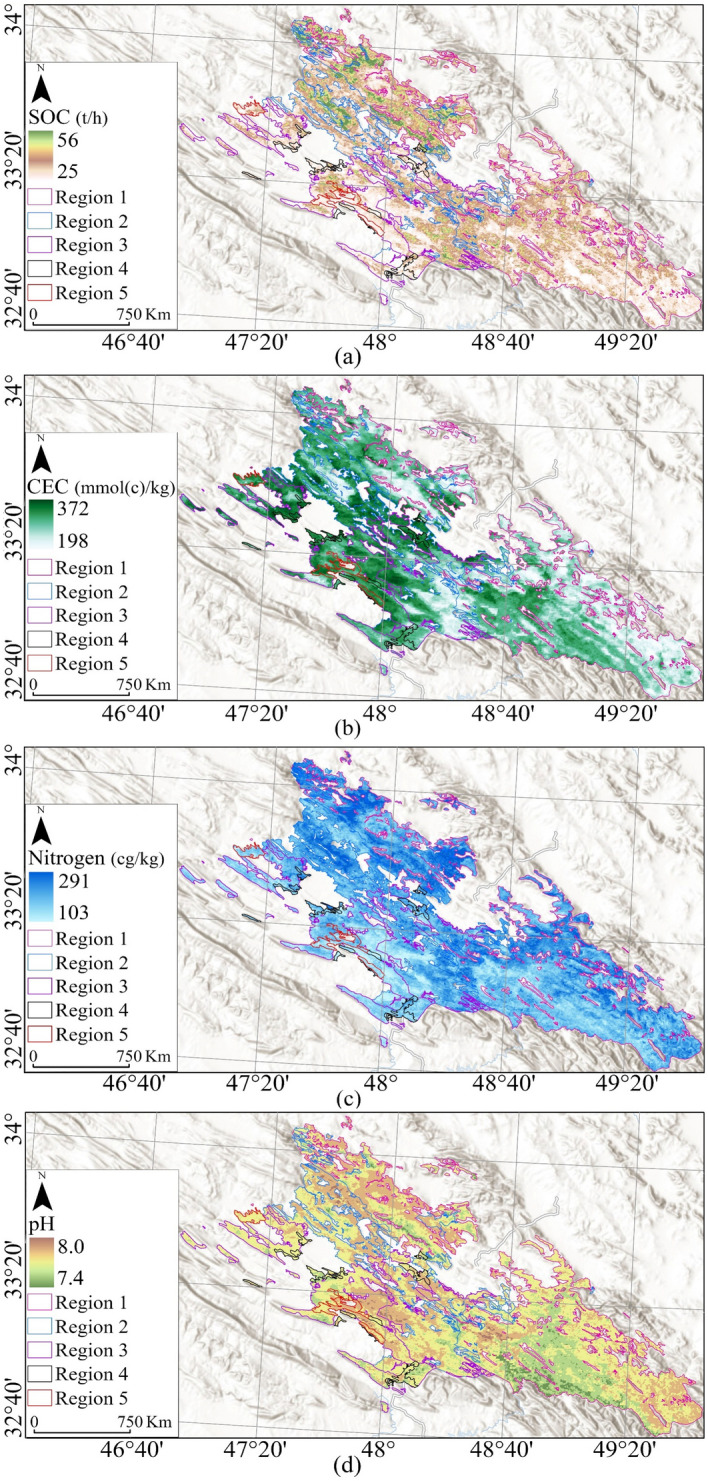
Soil chemical properties, SOC (**a**), CEC (**b**), Nitrogen (**c**), and pH, overlaying on National Geographic Style Map in Esri ArcGIS (version 10.8)^45^.

Eight, *soil physical and chemical properties*, coming from SoilGrids, produce maps of soil properties globally at an average spatial resolution of 250 m using machine learning methods^96,97^. Sand, silt, and density (Fig. 4) as physical and CEC, Nitrogen, and SOC (Fig. 5) as chemical properties were processed aligning with the boundary of each oak decline region.

### Method

The oak decline dataset divides the study area into five regions based on each region’s declined tree percentage. Considering these decline regions, we analyze abiotic factors, such as topographic features, land use classes, and long-term drought and metrological factors. The goal is determining which factor significantly affects each region’s oak decline.

#### Time series analysis

To decipher time-series data, this paper uses A Bayesian Estimator of Abrupt change, Seasonal change, and Trend (BEAST)^98^. A description of the BEAST method is provided in Appendix B. Furthermore, to identify hotspots in drought time series, i.e., periods with more than 25% severe and/or extreme drought, the changing points of the trend model are analyzed. This identifies points in which the overall behavior of drought is changed. Then, aggregating drought conditions between these changing points according to PDSI categories (Appendix C, Table C1) will identify more extended periods with each region's severe drought condition.

#### Metrological factors

The oak decline can be exacerbated by a precipitation deficit^27^. Therefore, this paper calculates the area under the precipitation graph to determine the total precipitation received by each region using a trapezoidal rule (Appendix D)^99^. Further, linear trends in metrological data are calculated to understand better the long-term behavior of the parameters and their probable future behavior. Linear trend estimation is a statistical technique to model the functional relationship between two or more parameters^100^. A description of the linear trend model is provided in Appendix E.

#### Topographical factors

Topography plays a crucial role in influencing the resilience of oak trees to climate change^10,15^. Consequently, this paper compares topographic features, such as elevation and slope, of regions with different decline percentages to determine how topography influences oak decline in the region. Thus, the frequency of elevation and slope of five decline regions, summarized in Table 1, are evaluated. This will provide an approximate representation of the distribution of the parameters^101^.

To calculate the frequency of elevation (and slope), the study area's elevation range (slope range) is divided into disjoint and equal intervals. The frequency is determined by counting the number of values within each interval. Also, the area of the regions is not equal, and frequencies are affected by the region’s size (Table 1). To remove the area effect from frequencies, they are normalizing using Eq. (1).

1 $$Normlized\, frequency= \frac{{f}_{i}}{{\sum }_{i=1}^{n}{f}_{i}}$$

where the *f*_*i*_ is the frequency of the i-th interval, and the ${\sum }_{i=1}^{n}{f}_{i}$ is the sum of frequencies of all intervals. The area effect is removed by normalizing frequencies.

## Results

### PDSI time series findings

Table 2 illustrates variations in drought conditions among regions from 1958 to 2022, with drought classes outlined in Appendix C, Table C1. Notably, Region 1 exhibited more favorable weather conditions compared to the other regions, with 58.5% of the duration falling within the normal and above-normal drought classes. Conversely, Region 5 faced the most severe drought conditions throughout the study period (1958–2022). Region 5 experienced droughts for 46.6% of the time, marking the highest drought occurrence among all regions.

**Table 2:** Properties of the PDSI index in five decline regions.

Drought class	Region 1		Region 2		Region 3		Region 4		Region 5	
	Count	%	Count	%	Count	%	Count	%	Count	%
Extremely drought	99	12.89	109	14.19	108	14.06	105	13.67	109	14.19
Severe drought	81	10.55	104	13.54	99	12.89	104	13.54	101	13.15
Moderate drought	139	18.10	137	17.84	142	18.49	138	17.97	148	19.27
Near Normal	325	42.32	300	39.06	302	39.32	302	39.32	293	38.15
Moderate wet	52	6.77	48	6.25	46	5.99	49	6.38	48	6.25
Very wet	30	3.91	31	4.04	27	3.52	24	3.13	29	3.78
Extremely wet	42	5.47	39	5.08	44	5.73	46	5.99	40	5.21

**Figure 6:**
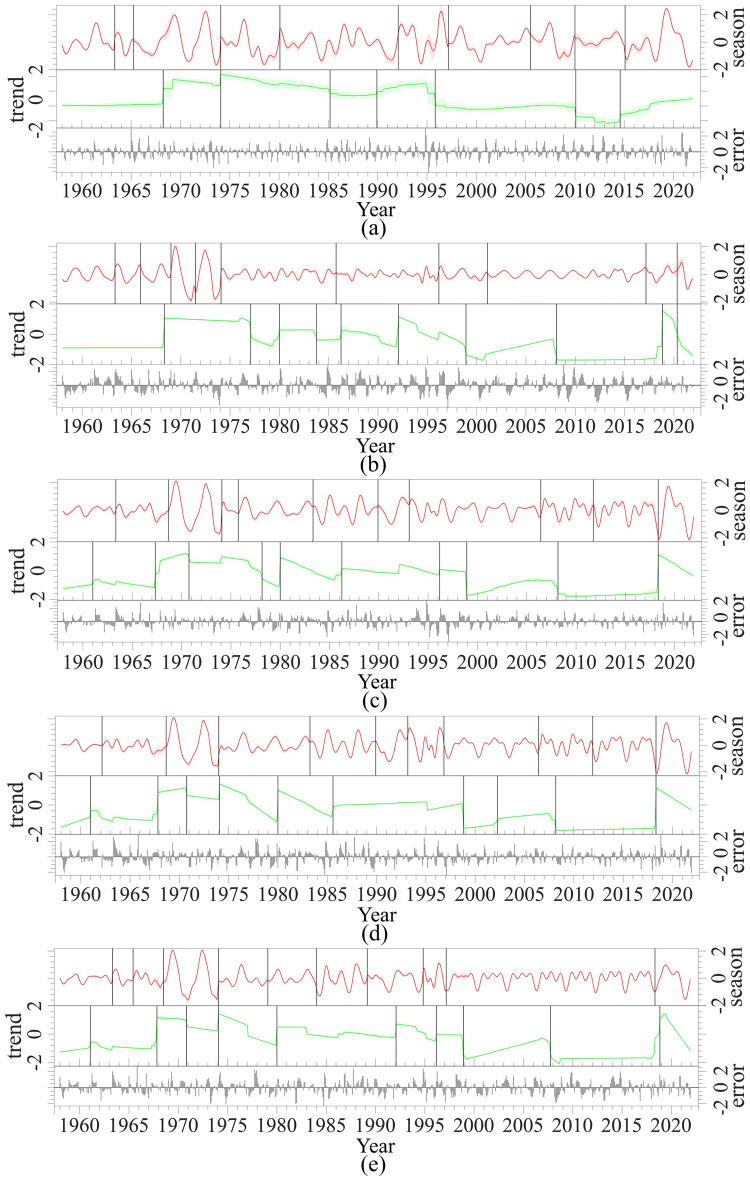
BEAST decomposition and changepoint detection of PDSI time series of Region 1 (**a**), Region 2 (**b**), Region 3 (**c**), Region 4 (**d**), and Region 5 (**e**); the black dots show the PDSI observations, Season is a piecewise harmonic model fitted to the seasonal component, Trend is fitted piecewise linear function, showing the trend of data, seasonal and trend changepoints are denoted by vertical black bars, and Error is the noise of decomposition process. Graphs are drawn using a Python package, Rbeast (version 0.1.14)^98,102^.

Furthermore, the PDSI time series of five decline zones is decomposed using the BEAST method. Figure 4a shows the decomposed PDSI time series of Region 1, which indicates that the PDSI time series of Region 1 has seasonal and trend components with no outliers. So, the fitted model matches Appendix B, Equation B1. In addition, the PDSI time series of Region 1 has a harmonic seasonal component with 80 months and ten changepoints with 99.9% probability. Furthermore, the trend component has seven changing points with a 33.2% probability. Changing points are shown with vertical black lines (Fig. 6). In September 1967, with 95% probability, the first change point in the trend component of Region one’s PDSI occurred, creating a 3.02 increase in PDSI value, with the highest variation in trend change points. Although the PDSI had positive values from September 1967 to January 1996, indicating normal and wet drought conditions (Table C1), it has a negative trend. This negative trend lasts until June 2014.

In Region 2, the PDSI has harmonic seasonal and trend components, as illustrated in Fig. 6b. So, the fitted model is ‘*Y* = *Trend* + *Season* + *Error*’. The seasonal components have ten changing points with a 99.9% probability. Also, the PDSI of Region 2 has 28 monthly seasonal components. Furthermore, a trend component has ten changing points with a 99.4% probability. All changing points are illustrated by black vertical lines in Fig. 5. The most significant change point in trend components happened in September 2018, which increased the PDSI value by 10.48.

The fitted model for the PDSI time series of Region 3 has seasonal and trend components (Fig. 6c). The fitted harmonic component has 37 month period. There is a 99.6% probability that the seasonal component has ten changing points. In addition, with a 97.4% probability, the trend components. In Region 3, in February 2018, the trend component had a significant change point, increasing the PDSI value by 4.78.

Also, the PDSI time series of Region 4 has seasonal and trend components (Fig. 6d). The harmonic model with a 37-month period is fitted to the periodic (seasonal) component. With a 99.9% probability, the fitted harmonic model for the seasonal component has ten change points. The trend component also has ten changing points. These points were identified with a 99.8% probability. The last change point with increasing PDSI by 6.85 was in January 2018.

Finally, Region Five’s PDSI time series also has trend and seasonal components (Fig. 6e). Its harmonic seasonal component has 36 month period. Ten change points were identified for the seasonal component with a 99.8% probability. The trend component showed ten change points with a 99.3% probability. Furthermore, in April 2018, there was a significant change in the trend model. This change point increased PDSI by 4.66.

**Figure 7:**
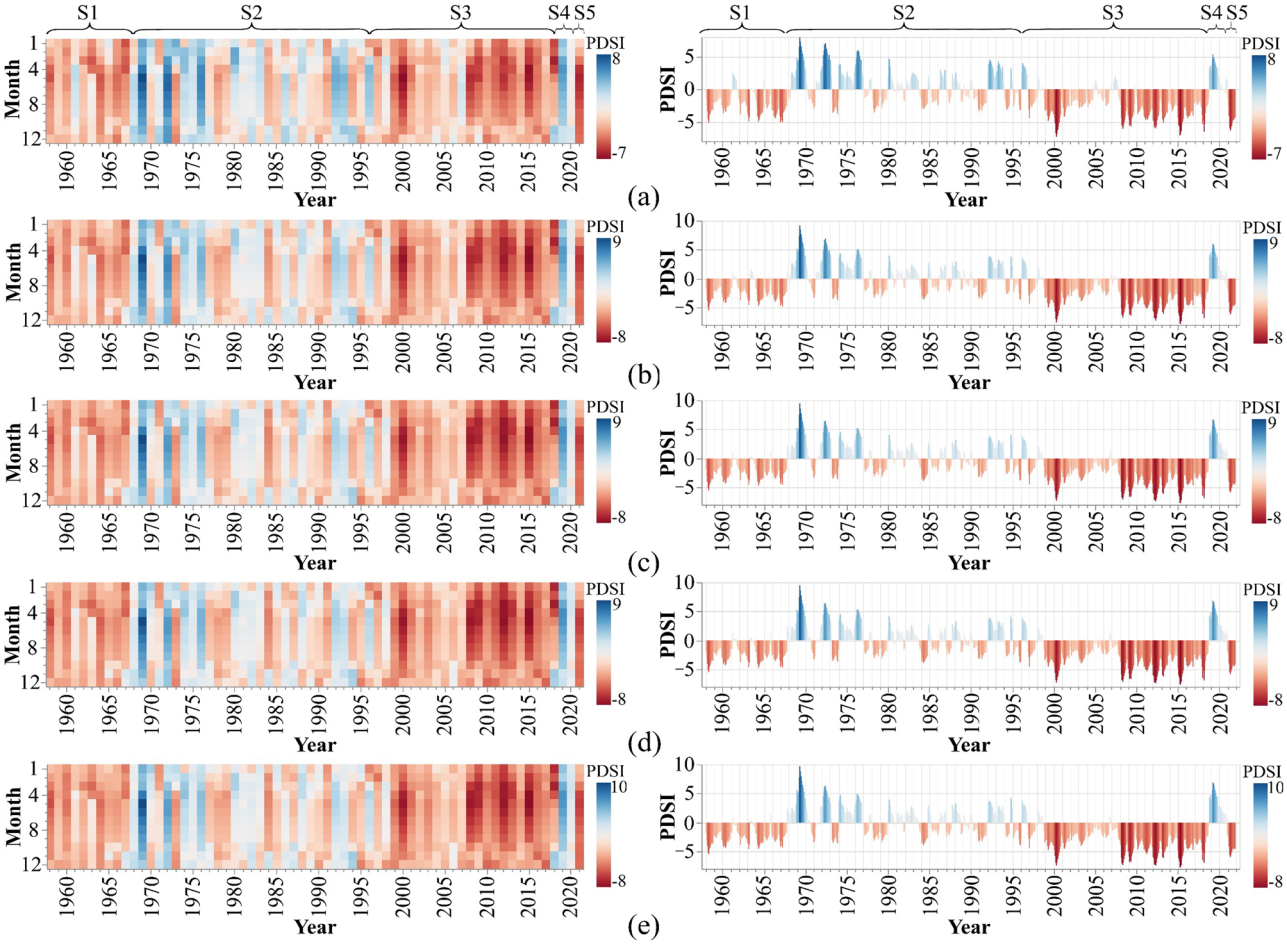
Time series of PDSI in decline regions: (**a**) Region 1, (**b**) Region 2, (**c**) Region 3, (**d**) Region 4, and (**e**) Region 5; The Altair, an interactive statistical visualization for Python (version 5.0.1), is used to draw graphs^103^.

**Figure 8:**
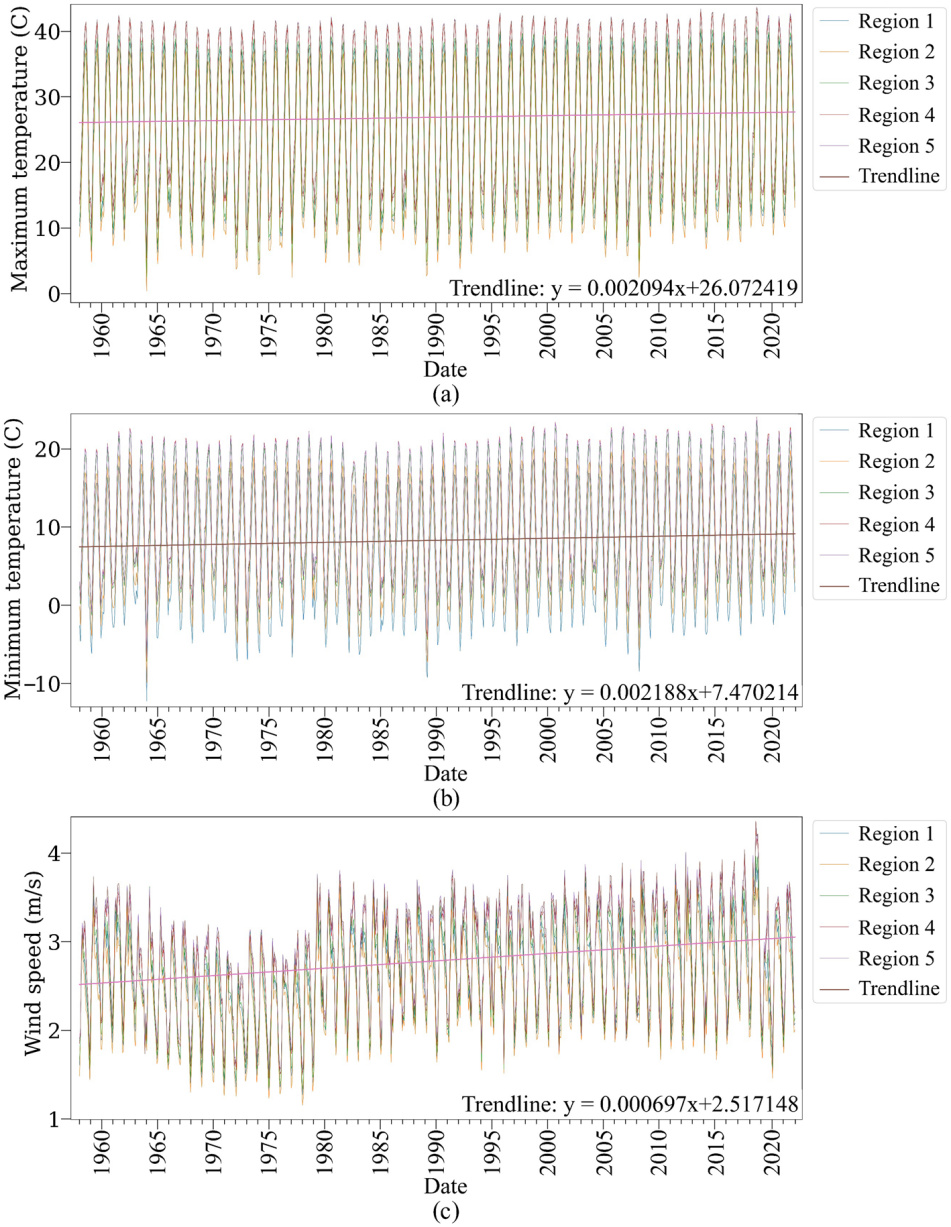
Time series of maximum (**a**), minimum (**b**) temperature, and wind speed (**c**) from 1958 to 2022; the Matplotlib for Python (version 3.7.2) is used to draw graphs^104^.

The analysis of the drought time series reveals four significant trend changes on specific dates: March 1968, November 1996, October 2018, and December 2020. These dates divide the study period (1958–2022) into five stages labeled S1 to S5 on the PDSI time series (Figs. 7 and 8). These trend changes indicate changes in drought behavior. Therefore, the drought behavior of each period (S1 to S5) is examined according to PDSI classification (Table C1).

During the S1 (June 1958 to March 1968), all regions experienced moderate to extreme drought (Table 3). Furthermore, less than 1% of the study area was characterized by a moderately wet climate. During these years, oak trees have been subjected to multiple drought stresses.

**Table 3:** Properties of the PDSI index from June 1958 to March 1968 (S1).

Drought class	Region 1		Region 2		Region 3		Region 4		Region 5	
	Count	%	Count	%	Count	%	Count	%	Count	%
Extremely drought	17	13.82	13	10.57	16	13.01	16	13.01	14	11.38
Severe drought	26	21.14	33	26.83	28	22.76	28	22.76	27	21.95
Moderate drought	31	25.20	31	25.20	37	30.08	36	29.27	42	34.15
Near Normal	47	38.21	46	37.40	41	33.33	42	34.15	39	31.71
Moderate wet	2	1.63	0	0.00	1	0.81	1	0.81	1	0.81
Very wet	0	0	0	0	0	0	0	0	0	0
Extremely wet	0	0	0	0	0	0	0	0	0	0

According to PDSI classification (Table C1), a near normal and wet climate (moderate, very, and extremely wet climate) was experienced in all regions during the S2 (April 1968 to November 1996). None of the five regions experienced extreme drought, while more than 80% of the regions fell into the normal and wet categories (Table 4). Additionally, 7.4% and 9.7% of this stage were extremely and very wet, respectively. Although better climate conditions provide a more favorable environment for forest growth, there were multiple drought stresses.

**Table 4:** Properties of the PDSI index from April 1968 to November 1996 (S2).

Drought class	Region 1		Region 2		Region 3		Region 4		Region 5	
	Count	%	Count	%	Count	%	Count	%	Count	%
Extremely drought	0	0	0	0	0	0	0	0	0	0
Severe drought	17	4.93	21	6.09	20	5.80	21	6.09	21	6.09
Moderate drought	40	11.59	41	11.88	44	12.75	42	12.17	46	13.33
Near Normal	178	51.59	179	51.88	183	53.04	182	52.75	181	52.46
Moderate wet	48	13.91	46	13.33	39	11.30	42	12.17	40	11.59
Very wet	25	7.25	27	7.83	25	7.25	23	6.67	27	7.83
Extremely wet	37	10.72	31	8.99	34	9.86	35	10.14	30	8.70

In S3 (December 1996 to October 2018), the study area faced drought. Only 27% of these 22 years had normal drought conditions. Furthermore, PDSI values indicate that in less than 1% of this period, oak forests had wet conditions (Table 5).

**Table 5:** Properties of the PDSI index from December 1996 to October 2018 (S3).

Drought class	Region 1		Region 2		Region 3		Region 4		Region 5	
	Count	%	Count	%	Count	%	Count	%	Count	%
Extremely drought	72	27.38	86	32.70	82	31.18	80	30.42	85	32.32
Severe drought	38	14.45	50	19.01	51	19.39	54	20.53	53	20.15
Moderate drought	67	25.48	64	24.33	60	22.81	59	22.43	58	22.05
Near Normal	86	32.70	63	23.95	69	26.24	69	26.24	66	25.10
Moderate wet	0	0.00	0	0.00	1	0.38	1	0.38	1	0.38
Very wet	0	0.00	0	0.00	0	0.00	0	0.00	0	0.00
Extremely wet	0	0.00	0	0.00	0	0.00	0	0.00	0	0.00

In S4 (November 2018 to December 2020), all regions experienced normal to extremely wet conditions, which means 60% of this period was wet, and the remaining were normal. Additionally, region 5 had better climate conditions than the other regions, with 69.23% of the duration being wet and 38.46% being extremely wet. However, in the S5 (January to December 2021), the drought condition completely changed, and 75.38% of the S5 period, the study area had an extreme drought.

### Metrological factors findings

The time series of maximum temperatures indicated that regions 4 and 5 had faced higher maximum temperatures in all months of the observation period (Fig. 8a). In contrast, region one experienced lower maximum temperature. Moreover, all regions showed an upward trend, indicating maximum temperatures increased from 1958 to 2022.

The minimum temperature time series indicates that Region 1 has a lower minimum temperature than other regions (Fig. 8b). However, region 5 experienced a higher minimum temperature than other regions. Additionally, all regions' minimum temperature time series have shown increasing trends during the observation period.

On the other hand, Regions 5 and 4 experienced higher wind speeds, while Regions 1 and 2 experienced lower winds (Fig. 8c). Furthermore, all regions demonstrated an increasing wind speed trend over the study period.

**Figure 9:**
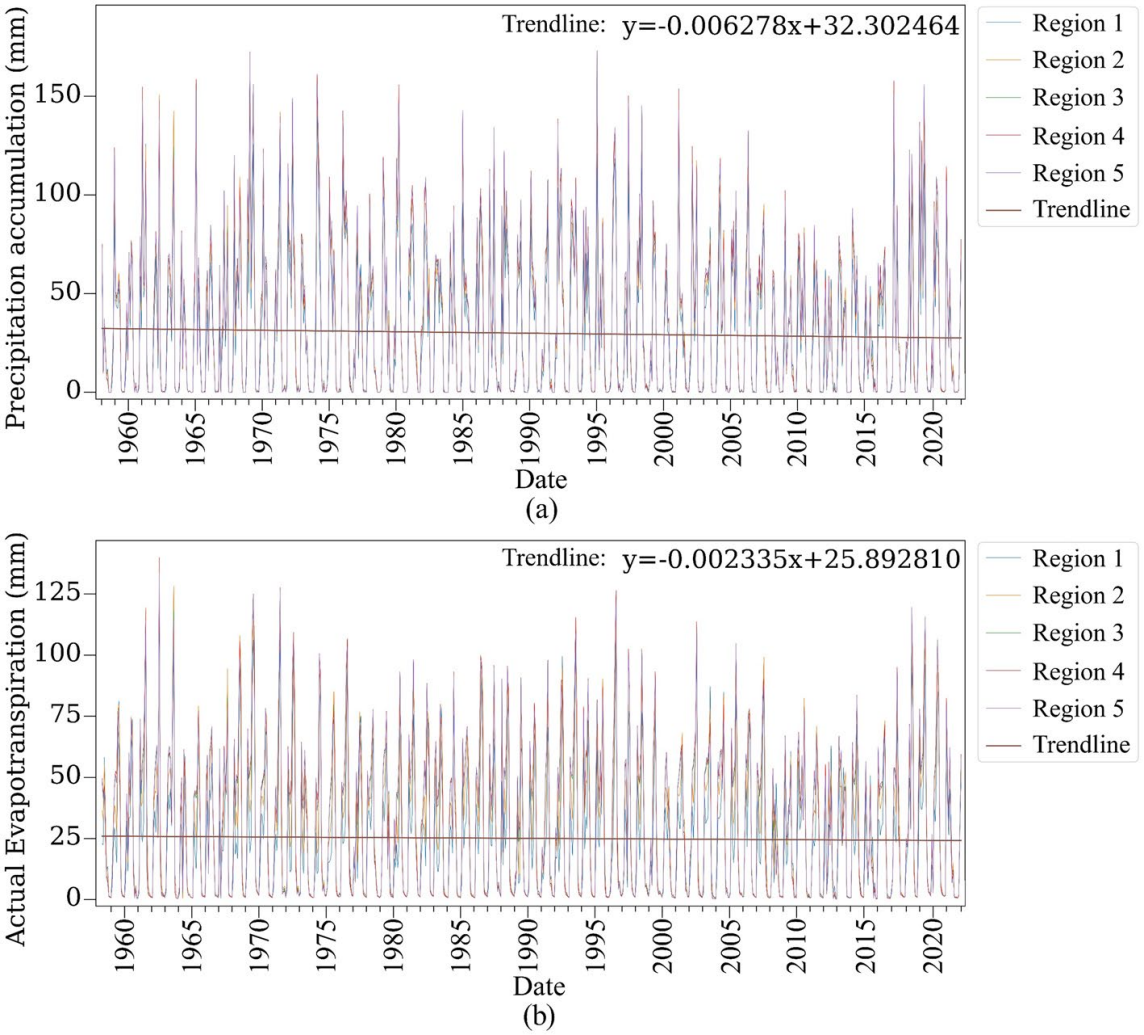
Normalized accumulated precipitation (**a**) and evapotranspiration (**b**) of five study regions from 1958 to 2022; the Matplotlib for Python (version 3.7.2) is used to draw graphs^104^.

The area of the regions is not equal (Table 1), and measurements are affected by the regions' size. Measurements are normalized to remove the area effect. Then, an area under the graph time series of normalized accumulated precipitation (Fig. 9a) and evapotranspiration (Fig. 9b) is calculated (Table 6). Results indicate that Region 4 had more accumulated precipitation from 1958 to 2022 (Table 6). However, Region 1 had the lowest accumulated precipitation. In addition, the evapotranspiration of Region 5 is more than other regions. Meaning that although this region received more precipitation, it had higher evapotranspiration.

**Table 6:** The area under the graph of normalized accumulated precipitation and evapotranspiration.

Region	Accumulated precipitation	Evapotranspiration
Region 1	22,895.1	94,014.8
Region 2	26,117.0	102,531.8
Region 3	26,776.2	112,853.8
Region 4	27,043.1	114,818.8
Region 5	26,923.2	114,909.5

### Topography findings

**Figure 10:**
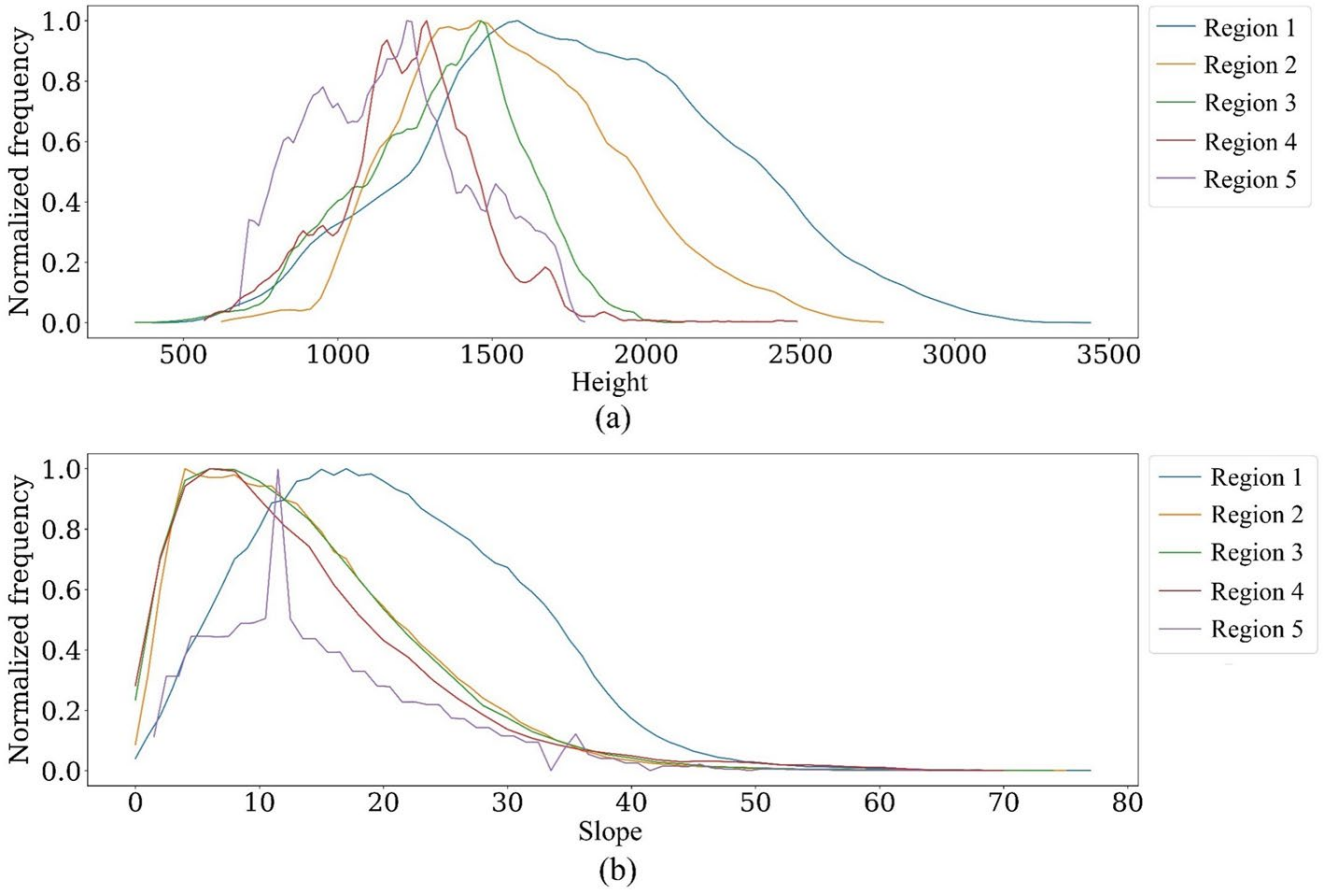
Normalized frequency of elevation (**a**) and slope (**b**) in the study regions**,** the Matplotlib for Python (version 3.7.2) is used to draw graphs^104^.

The normalized frequency of elevation in oak forests shows that Region 1 has the highest elevation in the study area, with most parts exceeding an altitude of 1500 m (Fig. 10a). This means high elevation positively affects oak forests’ resilience to the decline phenomenon. Moreover, most parts of Region 1 have slopes greater than 15%, which indicates that this region has steeper slopes than the other regions (Fig. 10b).

**Figure 11:**
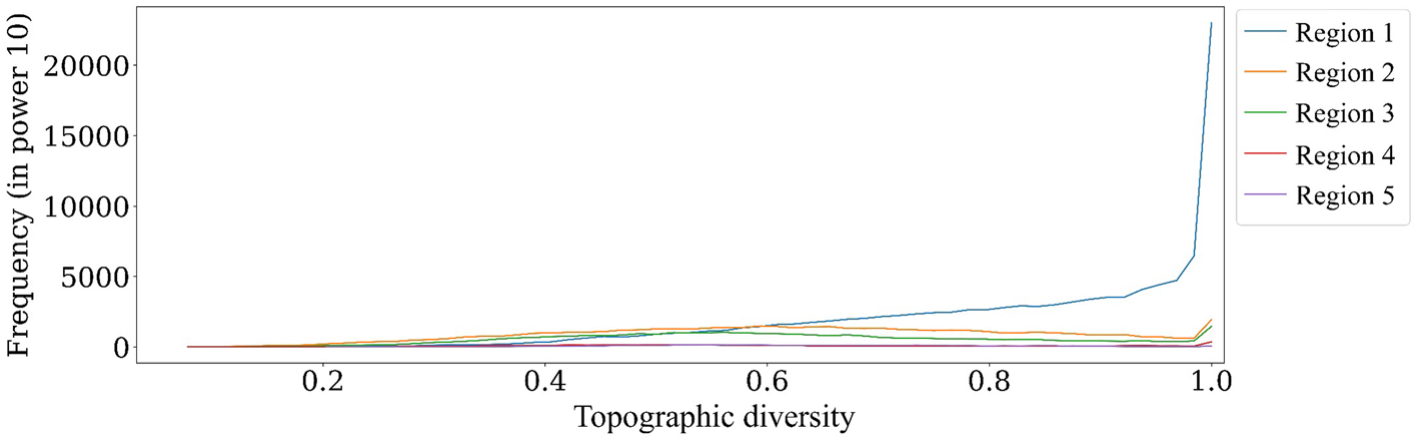
Regarding the frequency of topographic diversity in decline zones, the Matplotlib for Python (version 3.7.2) is used to draw graphs^104^.

Region 1 exhibits a distinct advantage in terms of topography, providing more favorable temperature and moisture conditions for oak growth compared to the other regions. This distinction highlights a significant disparity in topographic diversity between Region 1 and the remaining regions (Fig. 11). The unique topographic features of Region 1 likely play a crucial role in creating a conducive environment for oak development.

### Landuse findings

**Figure 12:**
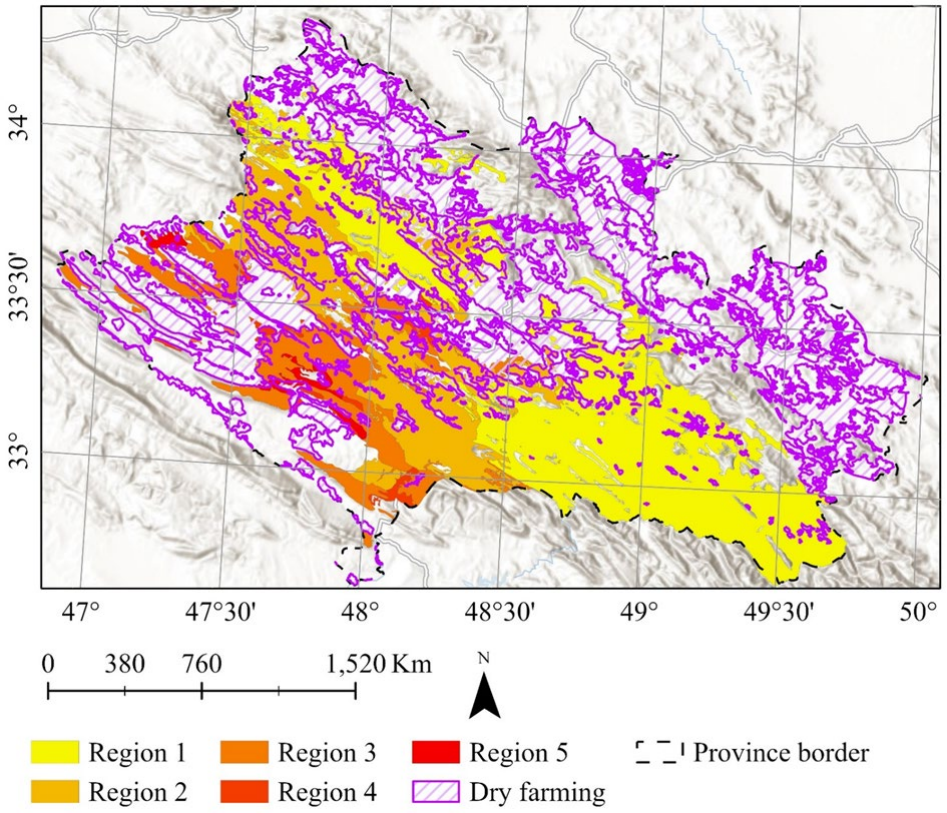
Overlay of dry farming lands with decline zones in Zagros oak forests of Lorestan province, overlaying on World Hillshade base map in Esri ArcGIS (version 10.8)^45^.

Most declining trees are concentrated in areas adjacent to dry farming lands (Fig. 12). Notably, the regions with the longest shared border with arid farming zones were identified as Regions 5 and 4. These findings underscore the potential influence of land use patterns, particularly the proximity to dry farming areas, on the occurrence and extent of oak decline within the study area.

### Soil data analysis findings

The physical and chemical characteristics of soil in the study area are summarized (Table 7). The soil has a high bulk density in most parts of Regions 3, 4, and 5 (Fig. 4a). In contrast, Region 1 has the lightest soil bulk density, with a mean density of 136.9 g/cm^3^ (Table 7). This means that high bulk density negatively affects oak trees and can increase oak decline.

**Table 7:** Statistical description of soil physical properties.

Bulk density (g/cm^3^)					Clay (g/kg)				Sand (g/kg)				Silt (g/kg)			
Region	Mean	Std	Skewness	Kurtosis	Mean	Std	Skewness	Kurtosis	Mean	Std	Skewness	Kurtosis	Mean	Std	Skewness	Kurtosis
One	136.9	2.5	0.02	3.75	369.8	36.6	− 0.28	2.70	208.9	48.2	0.28	2.63	421.3	28.2	0.04	2.95
Two	138.4	2.7	0.28	2.75	370.2	39.1	− 0.22	2.65	195.6	45.1	1.01	3.6	434.2	30.3	− 0.12	2.85
Three	140.2	2.8	0.12	2.46	338.2	32.7	0.07	2.90	235.9	49.3	0.40	2.17	425.9	34.2	− 0.08	2.20
Four	140.5	3.1	0.03	2.32	328.7	31.0	− 0.05	2.45	248.6	48.3	0.11	2.35	422.7	30.9	0.03	2.37
Five	140.3	2.5	0.73	3.0	331.5	41.5	0.07	2.10	269.3	56.6	0.10	1.83	398.8	28.2	0.57	2.90

According to the clay map (Fig. 4b), most parts of Regions 1 and 2 have clay soils. These two regions have about 30% more clay than other regions. This highlights the positive effects of clay soil on oak tree growth as these two regions have lower decline rates than others.

Regarding sand, Region 5 has the most sandy soil, with a mean of 269.3 g/kg of sand. Also, the southern parts of the whole study area have more sand than the northern parts (Fig. 4c). This underscores the potential side effects of sandy soils in oak decline. Region 2 exhibited the highest silt (434.2 g/kg), while Region 5 had less silt than others. Also, the north and northeast of the study area have more silt (Fig. 4d).

Analysis of soil’s chemical properties indicates that soils of Region 1 and 2 have the highest amount of SOC (Table 8), underscoring the positive effects of SOC in oak growth. In the case of soil’s CEC, all regions seem to have an acceptable amount of CEC. Also, Region 5 has a better condition with the highest mean CEC (317.7 mmol(c)/kg) than the others.

**Table 8:** Statistical description of soil chemical properties.

SOC (t/ha)					CEC (mmol(c)/kg)				Nitrogen (cg/kg)				pH			
Region	Mean	Std	Skewness	Kurtosis	Mean	Std	Skewness	Kurtosis	Mean	Std	Skewness	Kurtosis	Mean	Std	Skewness	Kurtosis
One	40.1	3.7	− 0.10	3.7	281.9	25.2	− 0.11	2.4	179.5	22.9	0.31	2.76	7.70	0.85	− 6.1	531.2
Two	40.7	3.7	− 0.12	3.9	301.0	27.5	− 0.46	2.3	175.1	23.4	0.05	2.94	7.74	0.63	− 0.22	3.10
Three	38.3	3.1	− 0.13	3.5	313.7	19.4	− 0.80	3.64	157.2	18.3	0.44	0.44	7.72	0.65	− 0.03	3.20
Four	37.9	3.3	− 0.03	4.5	316.8	16.7	− 0.69	3.56	162.6	22.0	0.46	3.4	7.71	0.57	− 0.03	3.10
Five	37.5	3.0	0.31	3.7	317.7	13.5	− 0.49	2.9	152.5	14.8	0.35	3.10	7.73	0.57	− 0.13	2.35

In addition, Region 1 has the most significant amount of nitrogen, with a mean of 179.5 cg/kg. Most parts of this region (Fig. 5c) have more than 148.8 cg/kg of nitrogen. Region 2 also has a high amount of nitrogen, while the soil in Regions 3 and 5 has less nitrogen.

Almost all study areas have a pH ranging from 7.4 to 8.8 (Table 8). Such a pH range is considered alkaline pH and is suitable for the availability of most nutrients to the roots^105^. Regarding soil chemistry, the most suitable pH range for root growth development is 6–7.5^106^. The center and north part of the study area have a more significant pH, while the south of the study area has a lower pH, which is more suitable for oak growth (Fig. 5d).

## Discussion

Drought is one of the leading causes of oak decline in the study area. Analyzing long-term drought data (64 years from 1958 to 2022) indicated that drought can significantly contribute to oak decline. Aligning with this finding, López-Sánchez et al.^14^, Capretti and Battisti^107^, and Macháčová et al.^27^ have identified drought as the leading cause of oak seedlings’ mortality. In the context of Zagros oak forests, aligning with our finding, Mahdavi et al.^108^, Ghanbary et al.^79^, Zolfaghari et al.^109^, and Jafarnia et al.^110^ found that drought is one of the leading causes of oak decline causes by increasing the aggressiveness of fungi and intensification of their destructive effects, making oaks oak seedlings more vulnerable to abiotic stressors.

The findings show that the overall trend of the PDSI data is negative, indicating that the region is facing more severe drought conditions towards the end of the study period according to PDSI classification (Table C1). This is aligned with the findings of Nazaripour et al.^111^, who reported that the frequency of more severe droughts has increased between 10 and 20 percent.

We found that droughts with 36 and 37-month seasonal periods can significantly contribute on oak decline. Because in regions 3, 4, and 5 where more than 25% of oaks are declined, we observed a 36 and 37-month seasonal components in drought time series. This is aligned with the findings of Safari et al.^112^, showing a more robust growth–drought association between 36- and 48-months of drought, which is aligned with our finding.

In addition, results showed that the region’s annual precipitation has decreased over the study period. We have discovered that adverse weather conditions, including increased temperatures, annual precipitation deficits, and high wind speeds, worsen oak decline in the study area. These conditions could also intensify biotic stresses. Notably, Region 5, characterized by such unfavorable weather conditions, exhibited a higher percentage of oak tree decline. Also, all these factors, temperatures, annual precipitation shortage, and wind speed, have negative trends during the study period (1958–2022). Aligning with this, Attarod et al.^113^, Barani and Karami^114^, Mahdavi et al.^108^, Rostamian et al.^115^, Safari et al.^112^, Poursartip et al.^116^, Henriques et al.^117^, Ghanbary et al.^54^, and Shiranvand and Hosseini^44^ highlighted effects of adverse weather conditions, e.g., decrease in precipitation, increase in evapotranspiration, temperature, and wind speed contributes to oak decline by facilitating transmission of some biotic factors, e.g., fungal, modifying the biochemical processes of trees, weakening and making them more vulnerable to biotic factors.

Our findings show that topographic factors, i.e., higher elevation, slope, and topographic diversity, enhance oak resilience against climate change. On the contrary, Dezfoli et al.^118^ reported divergent results. They found that slope does not significantly impact the oak decline rate. However, they observed higher oak decline in higher elevations. About 76% of their study has less than a 15% slope with little variability. On the other hand, the Zagros oak forest falls into a wide range of slopes, as shown in Fig. 10b. Hence, the findings of Dezfoli et al.^118^ are influenced by their choice of the study area. Thus, it becomes apparent that a more extensive study area should be considered when investigating complex phenomena like oak decline.

We have found that the proximity of oak woodlands to specific land use classes, such as dryland farming, plays a crucial role as an amplifier of oak decline. Regions 4 and 5, which encompass dryland farming areas, showcase a higher number of declined trees. These land use classes have direct and indirect effects on exacerbating oak decline. The direct impact involves the destruction of acorns and young seedlings due to plowing activities in rainfed farming and livestock grazing. Indirect intensification of the oak decline stems from an increase in human-forest interactions. In alignment with this finding, Pourhashemi^119^, Shiranvand and Hosseini^44^, Pourhashemi et al.^120^, Safari et al.^112^, Ghanbari Motlagh et al.^121^, Karami et al.^122^, Mausolf et al.^123^, and Soleymani et al.^124^ emphasized the importance of land use in vicinity of oak woodlands. They indicated that some land use classes, e.g., livestock grazing, fuel production, farming, and tourism activities, partially influenced the oak trees' sensitivity to biotic and abiotic extremes.

We have found that soil bulk density affects oak decline, and soils with lower density (Region 1) create a better environment for oak growth because Region 1, having lower bulk density, has fewer declined trees. This is aligned with Kormanek et al.^125^, indicating that soil bulk density significantly impacts the oak’s root system development, total height, and dry mass of the oak seedlings. Specifically, as soil density increased, the root system size decreased, affecting the proper tree development and stability. Even slight increases in soil compaction negatively affected young seedling growth. In contrast, areas characterized by high bulk density are situated adjacent to dry farming lands. Particularly, regions sharing the longest borders with arid farming zones, namely Regions 5 and 4, exhibit the highest bulk density. These findings underscore the potential influence of land use patterns, particularly the proximity to dry farming areas, on the soil bulk density and extent of oak decline within the study area. Aligning with the findings of Panagos et al.^126^, land use acts as the main driver for bulk density variation.

Therefore, sustainable forest management is crucial to address this issue, acknowledging the forest as a socio-ecological system encompassing ecological, social, and cultural aspects. Consequently, multidimensional studies are necessary to control and manage forest ecosystems effectively. Our recommendation for empowering oak forests to regrow, which is essential for ecosystem sustainability, involves assessing oak forest ecosystems from socio-ecological and land use policy management perspectives. This approach aims to identify more equitable and representative options for the sustainable management of the Zagros oak forests.

One potential outcome of conducting comprehensive studies and analyses to understand the effects of abiotic factors on oak decline in the study area could be the recognition that local communities perceive threats to the sustainability of oak forests. These threats may arise from increasing human-forest interactions, land use changes, economic development, and climate change-induced alterations in environmental conditions.

### Limitations

This study has a few limitations. First, we did not have access to data on biotic factors, making it essential to investigate the effects of both biotic and abiotic factors in oak decline simultaneously. Second, this paper did not examine uncertainty indices or datasets, which opens the possibility for future research to explore uncertainty by comparing the magnitudes of each source of uncertainty. Moreover, according to Vicente-Serrano et al.^127^, short drought time scales are primarily related to soil water content, while longer time scales are associated with variations in groundwater storage. As this study focuses on a long-term drought in the Zagros oak forests and does not assess groundwater data in the study area, it is recommended for future research to investigate the effects of changes in the groundwater table in the region. Additionally, exploring both spatial and temporal variations of abiotic factors in the Zagros oak forest is recommended to determine large-scale spatial variations of these parameters and their correlation with oak decline.

In addition, the Zagros oak forests consist of the western and southern slopes of the Zagros Mountains, which run from northwest to southeast, from the Turkish border to the Persian Gulf^38,128,129^, meaning that the climate conditions differ from north to south of Zagros oak forests. In the study, we could not find ground samplings of oak decline in the Zagros region. So, we limited the study area of this research to oak woodlands of central Zagros oak forests located in Lorestan province. Therefore, in a general study, investigating the effects of abiotic factors in the whole part of Zagros oak forest is recommended for future research.

In addition, as dust storms have become frequent in the west of Iran and the probable effects of dust on vegetation^130,131^, investigating the effects of air dust on oak decline is recommended for future study. Furthermore, deforestation and forest fires in the region could also be included as abiotic factors for forest decline since the region faces forest fires every summer.

### Supplementary Information

Supplementary Information.

## Data Availability

Datasets are publicly available via GitHub: https://github.com/smehri/Forest_Monitoring.

## Code availability

All Python codes used and/or analyzed during the current study are publicly available via GitHub: https://github.com/smehri/Forest_Monitoring.
