# Supplementary material for: Abiotic factors impact on oak forest decline in Lorestan Province, Western Iran
Source: Sci Rep. 2024 Feb 17;14:3973. doi: 10.1038/s41598-024-54551-6 (PMC10874411; doi:10.1038/s41598-024-54551-6)
Supplement: Supplementary file 1 — Supplementary Information. [file 41598_2024_54551_MOESM1_ESM.docx]

## Supplementary information

### Appendix A. Topographic diversity

Our knowledge of plants' climate adaptation process is still in its infancy and remains relatively complex. However, this knowledge can potentially develop strategies for preserving ecological patterns and processes in the face of climate change ^132,133^. Topography plays a vital role in shaping hydrology, climatology, geography, ecology, and understanding biodiversity ^134,135^. Consequently, the topographic diversity index becomes a valuable tool for expressing the range of different temperature and moisture conditions available to species as local habitats. Following the logic that a greater diversity of topo-climate niches can support higher plant diversity and enhance species' resilience to climatic change, we calculate the Topographic diversity D using **Equation A1**. This index helps us understand the extent to which diverse habitats are available to species based on various temperature and moisture conditions.

| $D=1-\left( 1-\acute{T} \right)*(1-\acute{C})$ | (A1) |
| --- | --- |

where *T'* is a dominant control of soil moisture, used to measure hillslope position and calculated in multiscale. The C' is a multiscale continuous Heat-Insolation Load Index (CHILI) ^91^.

### Appendix B. Bayesian Estimator of Abrupt change, Seasonal change, and Trend (BEAST)

The BEAST decomposes a time series γ = [*t_i_*, *y_i_*]*_i_*_=1, 2, …,_ *_n_* into three components, (1) seasonality, (2) trend, and (3) abrupt changes, in addition to noise, which is formulated as **Equation B1** ^98^.

| $y_{i}=S\left( t_{i};\Theta_{s} \right)+T\left( t_{i};\Theta_{T} \right)+\varepsilon_{i}$ | (B1) |
| --- | --- |

where ε is niose, *S*(**•**) and *T*(**•**) are sessional and ternd components, respectively. *S*(**•**) and *T*(**•**) are general linear models ^136^. Accordingly, Θ*_S_* and Θ*_T_* encode abrupt changes in seasonal and trend signals, respectively. The BEAST approximates seasonal signal *S*(*t*) with a piecewise harmonic model, as defined in **Equation B2**. The *S*(*t*) divides time series into (*p* + 1) segments on intervals [ξ*_k_*, ξ*_k+1_*]_k = 0, …,_ *_p_*, where the ξ*_0_* = *t_0_* and ξ*_p+1_* = *t_n_* are starting and ending points time of series, respectively.

| $s_{t}=\sum_{l=1}^{L_{k}} \left[ a_{k,l}\cdot\sin\left( \frac{2\pi lt}{P} \right)+a_{k,l}\cdot\cos\left( \frac{2\pi lt}{P} \right) \right]; \xi_{k}\leq t<\xi_{k+1}, k=0, 1, \ldots, p$ | (B2) |
| --- | --- |

where *P* is the period of the seasonal signal, and *L_k_* is a segment-specific parameter showing the order of the harmonic model in the k-th segment. The knots ξ*_k_* is changepoints at which abrupt seasonal changes may occur ^98^.

Accordingly, the trend of the time series, *T*(*k*) is modeled as a piecewise linear function according to **Equation B3** with *m* knots at τ*_j_*, *j* = 1, 2, …, *m*. The *T*(*k*) divides the time series into (*m* + 1) intervals [τ*_j_*, τ*_j+1_*]_j = 0, …,_ *_m_*, where the τ*_0_* = *t_0_* is the starting and τ*_m+1_* = *t_n_* is the ending point of the time series. Change points in trend model τ*_j_* differ from the seasonal change points ξ*_k_* ^98^.

| $T\left( t \right)=a_{j}+b_{j}t \mathrm{for} \tau_{j}\leq t<\tau_{j+1}, j=0, 1,\ldots, m$ | (B3) |
| --- | --- |

where *m* and τ*_0_* are trend change points and their timings, respectively, the BEAST method was implemented in Python using the Rbeast package ^102^.

### Appendix C. Palmer Drought Severity Index (PDSI)

Most regions on Earth are prone to experiencing droughts due to climate variability ^137^. Drought, a stochastic natural hazard, is the persistence and intense negative moisture anomalies over an extended period, like a season or a year ^138^. It is a regional environmental disaster ^94^, which can happen in all climate zones ^139^. Droughts can negatively impact the availability and quality of surface and groundwater resources ^140^. Furthermore, it can lead to crop failure, reduce range productivity, and adversely affect socioeconomic factors ^141^.

During recent decades, several drought indices have been developed. PDSI is one of the most widely used drought indicators addressing two of the most challenging aspects of drought: the intensity of the drought event and its beginning and end times ^94^. The PDSI is computed based on four major factors: precipitation, temperature, soil moisture, and evapotranspiration ^142^, and is calssified into seven classes (**Table C1**). In terms of drought warning systems, it is one of the most sophisticated and accurate indices. Furthermore, in the case of long-term drought determination, the PDSI is both a comprehensive and an effective method ^138^. A PDSI provides a better indication of drought severity in areas where actual evaporation exceeds a 3% anomaly in precipitation ^143^.

Table C1. Palmer drought index categories

| No. | **Category** | **PDSI value** |
| --- | --- | --- |
| 1 | extremely drought | ≤ -4.0 |
| 2 | severe drought | -3.99 to -3 |
| 3 | moderate drought | -2.99 to -2 |
| 4 | near normal | -1.99 to 1.99 |
| 5 | moderate wet | 2.0 to 2.99 |
| 6 | very wet | 3.0 to 3.99 |
| 7 | extremely wet | 4.0 or more |

### Appendix D. Trapezoidal rule

To calculate the region under the graph of a function, *f*(*x*), defined on an interval [*a*, *b*], the trapezoidal rule approximates the region with trapezoids ^99^. The shaded region in **Figure D1** represents the area to be measured. To ward this end, the interval [*a*, *b*] is divided to an even number, *2n*, of subintervals. Each subinterval has a width of ∆*x* = (*b-a*)/*2n*. The cross pending points of the *x*-axis are *x_0_* = *a*, *x_1_* = *a* + ∆*x*, *x_2_* = *a* + 2∆*x*, etc., and the corresponding coordinates are *y_0_*, *y_1_*, *y_2_*, etc. The area, $A=\int_{a}^{b} f\left( x \right)dx$ is approximated by the area of six trapezoids according to Eqaution C1 ^99^.

| $A\approx\frac{\Delta x}{3}\left( y_{0}+2y_{1}+2y_{2}+\ldots+2y_{2n-1}+y_{2n} \right)$ | (A1) |
| --- | --- |


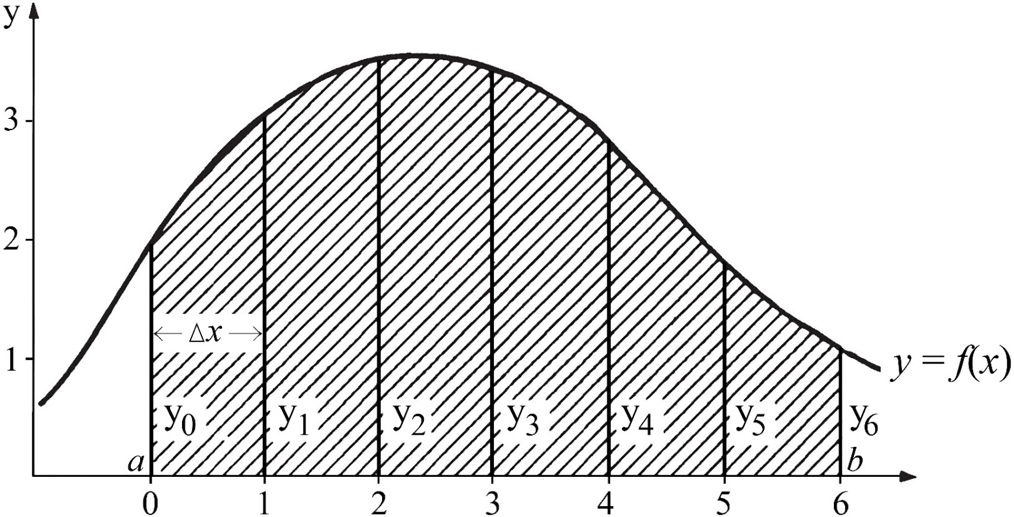


Figure D1. Numerical approximation of area using six subintervals ^99^

### Appendix E. Linear trend model

**Equation E1** defines a linear trend model which is fitted to data.

| $Ŷ= \beta_{0}+ \beta_{1}X+ \varepsilon_{i}$ | (E1) |
| --- | --- |

where *X* is an independent variable, e.g., wind speed. β0 and *β_1_* are the intercept and slope of the fitted linear model *Ŷ*, respectively. The *ε_i_* = *Y_i_ - Ŷ_i_* is residual, which has to be minimized. To minimize the sum of squares of residuals ∑(*Y_i_ - Ŷ_i_* )^2^, the Ordinary Least Squares (OLS) regression is used. Then, using OLS, *β_0_* and *β_1_* are calculated according to **Equations E2** and **E3**, respectively.

| $\beta_{1}= \frac{\sum_{i=1}^{n} \left( x_{i}-\overline{X} \right)\left( y_{i}-\overline{Y} \right)}{\sum_{i=1}^{n} \left( x_{i}-\overline{X} \right)^{2}}$ | (E2) |
| --- | --- |
| $\beta_{0}=\bar{Y}_{i}-\beta_{1}\overline{X}$ | (E3) |

where $\overline{X}$ and $\overline{Y}$ are the means of variables.
